# *Agrobacterium*-mediated transformation of rough lemon (*Citrus jambhiri* Lush) with yeast *HAL2* gene

**DOI:** 10.1186/1756-0500-5-285

**Published:** 2012-06-12

**Authors:** Shawkat Ali, Abdul Mannan, Mohamed El Oirdi, Abdul Waheed, Bushra Mirza

**Affiliations:** 1 Department of Biochemistry, Quaid-i-Azam University, Islamabad, Pakistan; 2 Département de Biologie, Université de Sherbrooke, Québec, Canada; 3 Horticulture R & D Centre Agriculture and Agri-Food Canada 430, Boulevard Gouin, St-Jean-sur-Richelieu, Québec, Canada; 4 Department of Pharmaceutical Sciences, COMSATS Institute of Information Technology, Abbottabad, 22060, Pakistan; 5 Department of Botany, University of Arid Agriculture, Rawalpindi, Pakistan

**Keywords:** Genetic transformation, Yeast halotolerant gene (*HAL2*), *Citrus jambhiri* Lush and *Agrobacterium tumefaciens*

## Abstract

**Background:**

Rough lemon (*Citrus jambhiri* Lush.) is the most commonly used *Citrus* rootstock in south Asia. It is extremely sensitive to salt stress that decreases the growth and yield of *Citrus* crops in many areas worldwide. Over expression of the yeast halotolerant gene (*HAL2)* results in increasing the level of salt tolerance in transgenic plants.

**Results:**

Transformation of rough lemon was carried out by using *Agrobacterium tumefaciens* strains LBA4404 harboring plasmid pJRM17. Transgenic shoots were selected on kanamycin 100 mg L^-1^ along with 250 mg L^-1^ each of cefotaxime and vancomycin for effective inhibition of *Agrobacterium* growth. The Murashige and Skoog (MS) medium containing 200 μM acetoseryngone (AS) proved to be the best inoculation and co-cultivation medium for transformation. MS medium supplemented with 3 mg L^-1^ of 6-benzylaminopurine (BA) showed maximum regeneration efficiency of the transformed explants. The final selection of the transformed plants was made on the basis of PCR and Southern blot analysis.

**Conclusion:**

Rough lemon has been successfully transformed via *Agrobacterium tumefaciens* with β-glucuronidase (*GUS)* and *HAL2.* Various factors affecting gene transformation and regeneration efficiency were also investigated.

## Background

Genetic transformation of *Citrus* is a valuable technique for *Citrus* improvement due to difficulties of conventional *Citrus* breeding. In *Citrus*, gene transformation is carried out by three different techniques i.e., particle bombardment [1] protoplast transformation [2] and *Agrobacterium*[3-7]. *Agrobacterium* mediated transformation is the most commonly used method for gene transfer to *Citrus* as it does not need embryogenic calli and is protoplast independent [2]. *Agrobacterium* mediated gene transformation have been reported for a number of *Citrus* species by different groups [8-10]. However the transformation efficiency is still relatively low for some *Citrus* species and is cultivar dependent [11-13]. Rough lemon (*C. jambhiri* Lush.) originated from Himalayan foothills in India and Pakistan, is the commonly used *Citrus* rootstock in riverland of South Australia, lemon growing area of Arizona [14], south Asia, and in Pakistan it is used as rootstock for more than 90% of citrus fruit plants. Rough lemon is very vigorous rootstock and produce high yield performance in early years [14]. Besides this it propagate quickly, produces well developed root system and result in producing large tree [14]. Its resistance to Tristeza virus and drought tolerance together with other quality mentioned make it rootstock of choice in citrus growing areas of Pakistan. On the other hand rough lemon is extremely sensitive to salt stress which is the inherited problem in irrigated soil. Besides this it is also susceptible to blight, alternaria leaf spot (*Alternaria citri*) and to foot rot (*Phytopthora parasitica*). To overcome these problems genetic transformation of rough lemon is very important for future of *Citrus* production. We have previously reported micropropagation of rough lemon [15] which is a prerequisite for *Agrobacterium* mediated transformation. The objective of this study was to evaluate the effect of different factors on gene transformation of rough lemon (*C. jambhiri* Lush.*)* in order to optimize the protocol that could be used routinely for genetic improvement, and also to transfer yeast *HAL2* gene to produce salt tolerant transgenic plants.

## Results and discussion

### Optimization of selection condition

**Figure 1:**
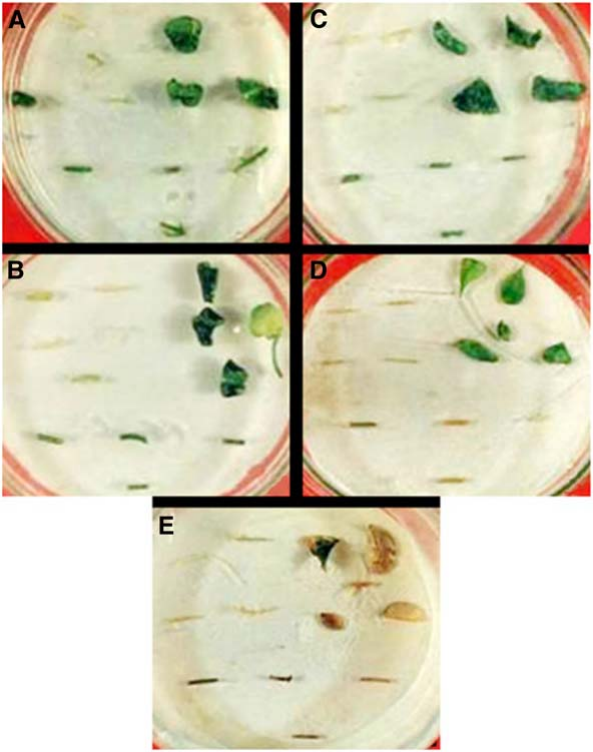
**Sensitivity of the untransformed explants (0.5-1 cm pieces of leaves and stem segments) were tested by putting explants in Petriplates in MS medium supplemented with kanamycin at concentration of (A) 0 mg L**^**-1**^**, (B) 25 mg L**^**-1**^**, (C) 50 mg L**^**-1,**^**(D) 100 mg L**^**-1,**^**and (E) 200 mg L**^**-1**^**.** The plates were incubated in growth chamber at 27°C, 16 h of photoperiod, illumination of 45 μE m^-2^ s^-1^ and 60% relative humidity for 14 days. The pictures were taken 2 weeks post incubation in MS medium.

For efficient and reliable production of transgenic *C. jambhiri* plants, optimization of the most suitable selection conditions is essential. The addition of a selective agent like kanamycin in the cultured medium is beneficial for competition of transformed cells with non-transformed ones and to decrease the number of escapes. The explants (stem and leaf) of untransformed plants were cultured on MS medium supplemented with 3.0 mg L^-1^ of BA containing various concentrations of kanamycin (0, 25, 50, 100, 200 mg L^-1^). Shoot regeneration was inhibited at the concentration of 50 mg L^-1^ of kanamycin in stem as well as in leaf segments as shown in Table 1. Concentrations of 100 mg L^-1^ and above resulted in complete bleaching and death of stem explants while in leaf explants 200 mg L^-1^ of kanamycin result in complete death (Figure 1, Table 1). Therefore, 100 mg L^-1^ kanamycin was used for selection of the explants throughout this work. It has been reported that 50 mg L^-1^ kanamycin completely inhibited shoot formation in non-transformed explants in trifoliate orange and hence 100 mg L^-1^ kanamycin was used for effective selection of transformed explants [16]. Pena and his group used 100 mg L^-1^ kanamycin for selection of transformed plants of different species of *Citrus*[9,17]. Different genotypes of *Citrus* may be resistant to slightly different concentration of selective agents like kanamycin in this study. Therefore it is important to investigate the sensitivity of untransformed explants before conducting any experiment of transgenic plant production.

**Table 1:** Influence of kanamycin dose on regeneration of the non-transformed explants

**Kanamycin mg L**^**-1**^**in regeneration medium**	**Average(Percentage ± SE) of segments producing shoots**	**Standard deviation**
0	45 (62.5 ± 0.4282)	1.0488
25	36 (50 ± 0.2582)	0.6325
50	0.0 (0 ± 000)	0.0000
100	0.0 (0 ± 000)	0.0000
200	0.0 (0 ± 000)	0.0000

Note. Data represent the mean value of three independent experiments. 50–75 explants were used in each experiment.

### Effect of inoculation media on transient expression

In order to select the best inoculation media three different types of media were tested in the transformation experiment in this study; LB medium, hormone free MS medium and MS medium supplemented with 200 μM AS. None of the explants inoculated in LB or hormone free MS media produce kanamycin resistant shoots while 60% of the explants inoculated in MS medium with 200 μM AS produced kanamycin resistant shoots. Each experiment was repeated three times with a total number of 80 explants. In all following experiments MS medium supplemented with 200 μM AS was used as inoculation medium for transformation. It has been shown that the addition of 20 μM AS to the inoculation media increase the transformation efficiency of *Citrus paradisi*[10]. A similar increase in transformation efficiency has been reported for lentil *(Lens culinaris* M.) by addition of 200 μM AS to inoculation medium [18].

### Effect of co-cultivation media on transient expression

To select a suitable co-cultivation medium, four different types of media were used in the transformation experiments in this study, i.e. plain MS medium, MS medium supplemented with 100 μM AS, MS medium supplemented with 200 μM AS and MS medium supplemented with 200 μM AS and 0.2 mg L^-1^ 2,4-Dichlorophenoxyacetic acid (2,4-D). Transient expression efficiency of explants on these media are shown in the Table 2. The MS medium supplemented with 200 μM AS showed maximum transformation efficiency (44%). The MS medium supplemented with 0.2 mg L^-1^ 2,4-D and 200 μM AS and MS medium supplemented with 100 μM AS showed transient expression efficiency of 26% and 25% respectively. The lowest transient expression efficiency was observed on plain MS medium. AS a plant phenolic compound produced in wound sites of plant, an inducer of the vir genes in *A. tumefaciens*[19]. The use of AS during co-cultivation has been shown to increase *Agrobacterium*-mediated transformation frequencies [20]. In carrizo citrange explants the addition of AS to co-cultivation medium increased the transformation frequency two fold [7]. Its beneficial role has also been demonstrated in transformation of some woody fruit, like apple [21] and kiwifruit [22]. Kaneyoshi *et al.*[16] reported the use of AS during co-cultivation of *Poncirus trifoliate* Rad. explants with *Agrobacterium;* however its role, as transformation enhancer was not investigated by them. The addition of certain auxins, especially 2,4-D, increase transformation frequencies in some *Citrus* species, (9,17). We conduct an experiment to investigate the effect of 2,4-D in combination with the 200 μM AS. Unexpectedly, the addition of this growth regulator in combination with AS decreased the transient expression efficiency. The possible explanation for this could, be that the addition of 2,4-D with AS decrease the transient expression but the efficiency of stable transformation will still be high. An alternative could be that as we transferred our explants after three days of co-cultivation into a medium that contains cytokinine instead of auxin as we want to get direct shoot regeneration without going to callusing and this could have an adverse effect.

**Table 2:** Influence of the co-cultivation media on transient expression efficiency of the rough lemon plants

**Types co-cultivation media**	**Average (Percentage ± SE) of segments producing shoots**	**Standard deviation**
Plain MS medium	5 (5.55 ± 0.3333)	0.5774
MS medium + 100 μM acetoseryngone	23 (25.55 ± 0.6667)	1.1574
MS medium + 200 μM acetoseryngone	40 (44.44 ± 0.6667)	1.1174
MS medium + 200 μM acetoseryngone + 0.2 mg L^-1^ 2,4-D	24 (26.66 ±0.5774)	1.000

Note. Data represent the mean value of three independent experiments. 75–100 explants were used in each experiment.

All media contains MS salts and vitamins.

### Effect of hormone concentration and combination on regeneration

To select a suitable medium for regeneration of putative transformed (kanamycin resistant shoots), three different types of media were used. All media used for regeneration were supplemented with 100 mg L^-1^ kanamycin sulphate for selection and either 500 mg L^-1^ cefotaxime alone or 250 mg L^-1^ cefotaxime and 250 mg L^-1^ vancomycin in combination for control of bacterial growth. Regeneration efficiency of explants on MS medium supplemented with 3 mg L^-1^ of BA was 40% and on MS medium supplemented with 2 mg L^-l^ BA and 0.1 mg L^-1^ of Naphthalene acetic acid (NAA), was 30% while on hormone free MS medium it was only 1.6% as shown in the Table 3. MS medium supplemented with 3 mg L^-l^ of BA or with 5 mg L^-1^ of BA has been used for regeneration of different species of *Citrus* by several laboratories [17,24]. MS medium supplemented with 5 mg L^-1^ BA and 0.1 mg L^-1^ NAA was used for the regeneration of *P. trifoliate*[16], while Costa *et al.*[25] reported shoot regeneration on BA from 0.5-4 mg L^-1^ with the best at 2 mg L^-1^ for *Citrus paradisi* (Macf) epicotyl explants. For citrange the best regeneration was obtained at 5 mg L^-1^ of BA [7]. However the percentage regeneration response in all these reports is quite low. Based on our results we used MS medium supplemented with 3 mg L^-1^ for regeneration in further experiments. Again here the regeneration efficiency may be genotype or cultivar dependent and a careful investigation may be required before conducting transgenic plant production experiments.

**Table 3:** Influence of hormone concentration and combination on regeneration

**Medium**	**No of segments cultured**	**Average (Percentage ± SE) of segments producing shoots**	**Standard deviation**
Hormone free MS medium	60	1 (1.66 ± 0.500)	0.7071
MS medium + 3 mg L^-1^BA	90	36 (40 ± 1.00000)	1.7321
MS medium + 2 mg L^-1^BA + 1 mg L^-1^ NAA	90	27 (30 ± 0.5774)	1.0000

Note: All media contain MS salts and vitamins, Data represent the mean value of three independent experiments.

### Effect of antibiotics on control of *Agrobacterium* during shoot regeneration

To control *Agrobacterium* growth during shoot regeneration, 500 mg L^-1^ of cefotaxime alone or 250 mg L^-1^ cefotaxime and 250 mg L^-1^ vancomycin were used. Results showed that cefotaxime alone did not inhibit *Agrobacterium* growth. However cefotaxime in combination with the vancomycin effectively inhibited the *Agrobacterium* growth during shoot regeneration. In some experiments, *Agrobacterium* start its growth again when regenerated transformed shoots were transferred to fresh medium without vancomycin after 4 weeks and these regenerated shoots died due to *Agrobacterium* over growth. Therefore in further experiments, cefotaxime and vancomycin in combinations were used for two months during shoot growth. For effective control of *Agrobacterium* growth in regeneration medium, Kaneyoshi *et al.*[16] used cefotaxime 500 mg L^-1^, while other group used cefotaxime 250 mg L^-1^ and vancomycin 250 mg L^-1^ in combination [13]. However, after 4 week they did not use any antibiotic for *Agrobacterium* control while in our experiment *Agrobacterium* start its growth again when antibiotics were omitted. It seems that vancomycin and cefotaxime did not kill *Agrobacterium* at this concentration but restrict its growth, which grew again when there was no selection pressure. It may be the reason that in some other experiments, higher concentration of cefotaxime 500 mg L^-1^ and vancomycin 250 mg L^-1^ in combination has been used [17,24].

### Rooting of transformed plants

For rooting, the developed shoots cut off segments were cultured on MS medium supplemented with 0.5 mg L^-1^ NAA or 1 mg L^-1^ 2, 4-D. We obtained 70% and 50% rooting results in these two media respectively. Low rooting efficiency has been previously reported as major problem for *in vitro* production of *Citrus* plants [26]. Difficulties in inducing roots have been found in transformation procedures of tree species, like walnut [27], apple [28], plum [29] and carrizo citrange [30] and have resulted in low production of regenerated transgenic plants. Peña *et al.*[13] used MS medium supplemented with 3 mg L^-1^ NAA for rooting of sweet orange and got only 3.2% rooting after 3 months of transferring shoots to rooting medium. However, similar to our results, 81.1% rooting on MS medium supplemented with 0.5 mg L^-1^ NAA was reported for trifoliate orange [16].

### Histochemical Gus assay

**Figure 2:**
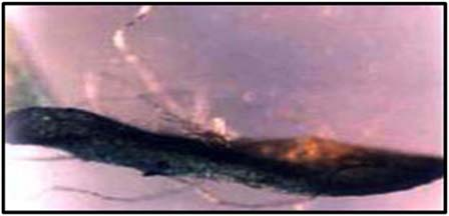
**Transient *GUS *expression in explants. ** Transient *GUS* gene expression in stem segment explants was observed immediately after co-cultivation with *A. tumefaciens* strain LBA4404/pJRM17.

The leaves and stem segments cocultivated with *Agrobacterium* strain LBA4404 harboring plasmid pJRM17 for three days were stained with *GUS* substrate by incubating for several hours at 37°C. Non-transformed explants showed no *GUS* activity when stained with (5-bromo-4-chloro-3-indolyl glucuronide (X-Gluc), while explants which were transformed showed blue spots (Figure 2).

### Molecular analysis of transformed plants

For molecular analysis of transformed plants, PCR and Southern blots were used to detect the transfer and integration of the Neomycin phosphotransferase (*NPTII)* and *HAL2* gene. Fifty-five kanamycin resistant (putative transformed) plants were produced with *HAL2* genes.

**Figure 3:**
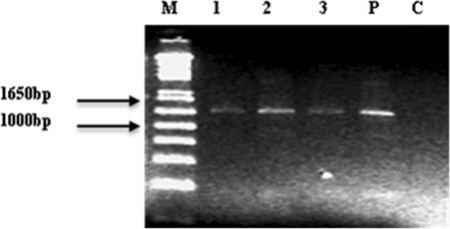
**A PCR analysis of *NPTII *gene in transgenic plants. ** Lane 1–3 represents independent *NPTII* positive plants resistant to kanamycin. Lane P corresponds to plasmid pJRM17 and Lane C to non-transformed control plantlet. Lane M 1 kb plus ladder Marker.

For PCR analysis DNA was extracted from healthy kanamycin resistant regenerated plants. A predicted internal fragment of the *NPTII* gene of about 1021 nucleotides was amplified in three individual plants as shown in the Figure 3. No amplification was found in samples from non-transgenic control plants. The same size fragment was also amplified from plasmid pJRM17.

**Figure 4:**
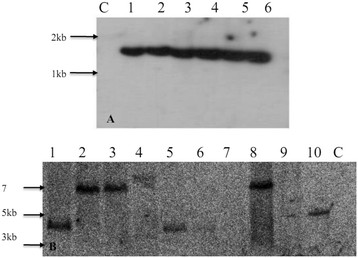
**Southern analysis of T-DNA integration in the transgenic plants.** Genomic DNA was digested with either HindIII (Figure 4A) or KpnI (Figure 4B). The membrane that contains HindIII digested DNA was probed with DNA fragments for *HAL2* gene located in the center of T-DNA (Figure 4A) and the other membrane that contains the KpnI digested DNA was probed with the DNA fragment for *GUS* gene located close to the RB (Figure 4B). (Figure 4A) Lane 1–6 independent *NPTII* PCR positive transformed plants C, negative control genomic DNA from the untransformed plants. (Figure 4B) Lane 1–10 independent *NPTII* PCR positive transformed plants C, negative control genomic DNA from the untransformed plants. The lane number from 1–6 in both Figure 4A and Figure 4B correspond to the same transgenic plants, while in Figure 4B four more transgenic plants were analyzed by southern blotting.

**Figure 5:**
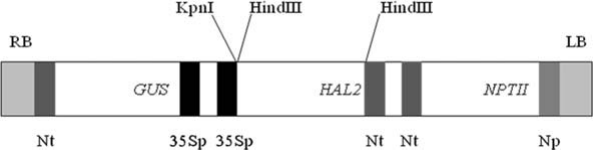
**Structure of the T-DNA region of the plasmid pJRM17.** The 7.4 kb DNA fragment between the right (RB) and left (LB) borders; Np: nopaline synthase gene promoter; Nt: nopaline synthase gene polyadenylation and transcription terminator site; 35 Sp: cauliflower mosaic virus 35 S promoter; GUS: β-glucoronidase coding region of the bacterial *gus* A gene; *NPTII*: neomycin phosphotransferase coding region of the bacterial *NPTII* gene; *HAL*2: fragment containing the coding region of the yeast *HAL*2 gene.

To determine the stability and randomness of T-DNA integration DNA was isolated from PCR positive transformed plants, and one untransformed plant as a negative control for Southern analysis. A single fragment of 1.6 kb, of expected size was observed for all six transgenic plants (Figure 4A) when an internal probe for the *HAL2* gene was used. This analysis confirm that all the transgenic plants were stably transformed, however it did not verify a single or random integration of the T-DNA as this was an internal probe for the T-DNA (Figure 4A). To see the randomness of the T-DNA integration and to confirm that these were independent transgenic plants, we performed another blot in which we used the probe for the *GUS* gene close to RB of the T-DNA and DNA was digested by restriction enzyme KpnI. As shown in the (Figure 5) each band in this blot should represent a single T-DNA insertion. At least five of the transgenic plants analyzed gave a different size junction fragment (Figure 4B) indicating a random integration of T-DNA in different parts of the genome. We did not see a clear visible band for two PCR positive transformants, which could be the result of T-DNA truncation at the right border and no band was observed in untransformed control plant. T-DNA truncation is common during *Agrobacterium* mediated transformation of plants but it is more common at right boarder than left border [31-33].

## Conclusion

The present research work results in successful transformation of *HAL2* gene in rough lemon using *Agrobacterium tumefaciens* strains LBA4404.

## Methods

### Preparation of explants

Seeds of *C. jambhiri* (Lush) were peeled removing both seed coats, disinfected in 0.1% (w/v) Mercuric Chloride for 5 min and rinsed thrice with autoclaved doubled distilled water. The seeds were then placed individually in culture tubes containing 25 ml of MS medium [34], containing 5% sucrose and solidified with 0.8% agar. The pH of all plant tissue culture media used in this study was adjusted to pH 5.75, with HCL and NaOH, while the pH of LB media was adjusted to 7.5. The media with the culture tubes was autoclaved before placing the seeds. For germination, the cultures tubes were incubated in darkness at 27°C for 2 weeks and then at 25°C, in growth chamber with 16 h of 45 μE m^-2^ s^-1^ photoperiod and 60% relative humidity for 3 weeks. Leaves and stem segments were excised from 5-week-old *in vitro* grown seedlings and cut into 0.5-1 cm pieces, which were used as explants for further manipulation.

### Binary vector and bacterial strain used

*Agrobacterium tumefaciens* strains LBA4404 [35] harbouring plasmid pJRM17 [36] was used for the transformation of rough lemon. The structure of the T-DNA region of pJRM17 carrying three chimeric genes is shown in the (Figure 5). The full description of plasmid JRM17 is describe by Arrillaga et al. [36]

### Preparation of inoculum

*A. tumefaciens* strain LBA4404 harboring plasmid pJRM17 was grown overnight in 50 ml liquid LB medium pH 7.5 containing 50 mg L^-1^ kanamycin sulphate and 30 mg L^-1^ rifampicine at 28°C in shaking incubator at 200 rpm. Bacterial cells were collected by centrifugation at 4000 rpm for 15 min and resuspended in inoculation medium. The density of bacteria was adjusted to approximately 5x10^8^ CFU ml^-1^.

### Transformation procedure

The stem and leaf explants were cut transversely from 5-week-old *in vitro* grown seedlings and then immersed in the bacterial suspension for 20 min in three different media (LB, plain MS and MS medium containing 200 μM AS). Thereafter the segments were blotted on sterilized filter paper and placed onto a co-cultivation medium in petri plates. Four different types of media were used for co-cultivation (plain MS medium, MS medium with 100 μM AS, MS medium with 200 μM AS and MS medium with 0.2 mg L^-1^ 2, 4-D and 200 μM AS). The co-culturing of explants was carried out in growth chamber at 27°C, 16 h of photoperiod, illumination of 45 μE m^-2^ s^-1^ and 60% relative humidity for 3 days.

After co-cultivation for three days, the explants were washed with sterilized MS medium containing either 500 mg L^-1^ cefotaxime alone or 250 mg L^-1^ cefotaxime and 250 mg L^-1^ vancomycin in combination, to control bacterial growth and blotted on sterilized filter paper. The segments were then transferred for shoot regeneration to the medium supplemented with 100 mg L^-1^ kanamycin sulphate for selection and either 500 mg L^-1^ cefotaxime alone or 250 mg L^-1^ cefotaxime and 250 mg L^-1^ vancomycin in combination for control of bacterial growth. Three different types of media were used for shoot regeneration (hormone free MS medium, MS medium supplemented with 3 mg L^-1^ BA, MS medium supplemented with 2 mg L^-1^ BA and 0.1 mg L^-1^ NAA). The cultures were maintained at 27°C, 16 h of 45 μE m^-2^ s^-1^ photoperiod and 60% relative humidity. After 4 weeks, segments with adventitious shoot were transferred to MS medium supplemented with 0.5 mg L^-1^ BA, 100 mg L^-1^ kanamycin sulphate, 250 mg L^-1^ cefotaxime and 250 mg L^-1^ vancomycin to allow further shoot development. For rooting, developed shoots segments were cultured on MS medium, supplemented with 0.5 mg L^-1^ NAA or 1 mg L^-1^ 2- 4-D.

### Histochemical Gus assay

β-glucuronidase (*GUS*) gene is the most commonly used reporter gene in plant transformation studies. Transient GUS expression was observed in leaf and stem segments after co-cultivation period of 3 days. The *GUS* assay was carried out according to the method of Jefferson *et al.*[37]. The leaf and stem segments were incubated in X-Gluc solution consisting of 1 mg L^-1^ X-Gluc, 0.5% triton X-100, 20% methanol and 50 mM NaH_2_PO_4_, pH 7, for several hours at 37^o^C. The chlorophyll was removed by several washes with 70% ethanol. Assayed tissues were observed under microscope.

### Molecular analysis

For the confirmation of transformation and integration of the *NPTII* and *HAL2* gene, molecular analysis was carried out through PCR and southern blotting.

For PCR analysis, genomic DNA was isolated from leaves of the transformed plants as well as untransformed plants by simplified CTAB method [38]. PCR was used to confirm the presence of *NPTII* gene (1021 bp) using the primers NPTIIF 5^’^-AAGATGGATTGCACGCAGGTTC-3^’^ and NPTIIR 5^’^-GAAGAACTCGTCAAGAAGGCGA-3’ following the standard protocol. The reaction conditions were as follows: 5 min of 95°C for template denaturation followed by 25 cycles of amplification of 30 s at 95°C; one minute at 55°C and one minute at 70°C and final extension of 10 min at 70°C. PCR was performed using Gene Amp PCR system 2400 thermocyclers (Perkin Elmer, USA).

For southern blotting, genomic DNA was extracted as described above. A 20 μg of total DNA was digested with either HindIII, or KpnI, separated by gel electrophoresis in 0.8% (w/v) agarose gel, in 1X Tris–borate–EDTA (TBE) buffer (89 mM Tris base, 89 mM boric acid, 2 mM EDTA pH8.0). The DNA from gel was transferred overnight to a Hybond-N + membrane as recommended by the supplier (Amersham Biosciences/GE healthcare). To label the probe, P32-dCTP was used, using the Rediprime II Random Prime Labeling system (AmershamBiosciences/GE healthcare). The membrane was hybridized at 42°C in the ULTRA-hyb buffer (Ambion) according to the manufacturer’s instructions. The blots were washed at 42°C, three time, 5 min each with 2 X SSC (0.3 M sodium chloride, 0.03 M sodium citrate, pH 7.0) and 0.1% (w/v) sodium dodecyl sulfate (SDS). Another two washes was carried out for 15 min each wash in 0.1 X SSC and 0.1% SDS. The blots were exposed to Kodak Biobax film (Kodak Canada, Toronto, ON, Canada).

## Abbreviations

BA: 6-Benzylaminopurine; CFU: colony Forming Unit; NAA: naphthalene acetic acid; 2,4 D:2,4-Dichlorophenoxyacetic acid; AS: Acetoseryngone.

## Competing interests

The authors declare that they have no competing interests.

## Author’s contribution

SA and AM performed the experiments, SA wrote the manuscript; SA, AW and BM conceived and designed the experiment and analyzed the data; MEO analyzed the data, BM provides reagents and materails, supervised the study and edits the manuscript.

## Author details

^1^Department of Biochemistry, Quaid-i-Azam University, Islamabad, Pakistan.^2^Département de Biologie, Université de Sherbrooke, Québec, Canada.^3^Horticulture R & D Centre Agriculture and Agri-Food Canada 430, Boulevard Gouin, St-Jean-sur-Richelieu, Québec, Canada. ^4^Department of Pharmaceutical Sciences, COMSATS Institute of Information Technology, Abbottabad 22060, Pakistan. ^5^Department of Botany, University of Arid Agriculture, Rawalpindi, Pakistan.
